# Eco-Friendly UPLC–MS/MS Method for Determination of a Fostamatinib Metabolite, Tamatinib, in Plasma: Pharmacokinetic Application in Rats

**DOI:** 10.3390/molecules26154663

**Published:** 2021-07-31

**Authors:** Essam Ezzeldin, Muzaffar Iqbal, Yousif A. Asiri, Ahmed Y. A. Sayed, Rashad Alsalahi

**Affiliations:** 1 Department of Pharmaceutical Chemistry, College of Pharmacy, King Saud University, Riyadh P.O. Box 11451, Saudi Arabia; muziqbal@ksu.edu.sa (M.I.); ahmedyahia@gmail.com (A.Y.A.S.); ralsalahi@KSU.EDU.SA (R.A.); 2 Bioavailability Unit, Central Laboratory, College of Pharmacy, King Saud University, Riyadh P.O. Box 11451, Saudi Arabia; 3 Drug Bioavailability Center, National Organization for Drug Control and Research, Cairo P.O. Box 29, Egypt; 4 Department of Clinical Pharmacy, College of Pharmacy, King Saud University, Riyadh P.O. Box 11451, Saudi Arabia; yasiri@ksu.edu.sa

**Keywords:** fostamatinib, tamatinib, UPLC–MS/MS, pharmacokinetics, rat

## Abstract

Fostamatinib is a prodrug of the active metabolite tamatinib, which is a spleen tyrosine kinase (Syk) inhibitor used in the treatment of primary chronic adult immune thrombocytopenia and rheumatoid arthritis. A highly sensitive, rapid, reliable, and green method was developed and validated using ultra-performance liquid chromatography and tandem mass spectrometry (UPLC–MS/MS) for quantification of tamatinib in rat plasma. Ibrutinib was used as internal standard and liquid–liquid extraction was applied using tert-butyl methyl ether. The analyte was separated on an Acquity^TM^ CSH C_18_ (2.1 mm × 100 mm, 1.7 µm) column using mobile phase consisting of 10 mM ammonium acetate and acetonitrile (10:90) and the flow rate was 0.25 mL/min. Electrospray ionization (ESI) was carried out in positive mode. Quantitation of tamatinib and the IS was performed using multiple reaction monitoring mode with precursor-to-product transitions of *m*/*z* 471.1 > 122.0 and *m*/*z* 441.1 > 84.0, respectively. The calibration range was 0.1–1000.0 ng/mL and the linearity of the method was ≥0.997. The developed method greenness was investigated. All principal parameters for the method, including linearity, accuracy, precision, recovery, and stability, were within acceptable ranges. Tamatinib pharmacokinetic study in rats was successfully carried out using the developed method.

## 1. Introduction

Fostamatinib is an oral Syk inhibitor that is effective in the treatment of malignant clones through inhibition of antigen-dependent BCR signals [1,2]. Fostamatinib is a prodrug of the active compound, tamatinib, which is a relatively selective Syk inhibitor used to treat rheumatoid arthritis [3,4], especially in patients with poor therapeutic response to methotrexate [5]. It is also used to treat thrombocytopenia, a shortage of blood platelets needed for normal blood clotting [6]. A recent study reported that mice treated with fostamatinib showed less fibrosis and inflammation in lung tissue [7]. Moreover, fostamatinib has a therapeutic option for limiting breast cancer metastasis [8] and for treatment of rheumatoid arthritis [9]. The drug can induce time- and dose-dependent decline in cell viability that is linked to apoptosis activation. This activity causes a reduction in tumor growth and was enhanced in combinations with other treatments, including bortezomib, rituximab, and dexamethasone [10]. However, dropout and subsequent loss of efficacy during fostamatinib treatment increased with the increases of its dose [3].

Fostamatinib administration shows variable effects on the pharmacokinetics of other drugs. Clinical investigations indicate that co-administration of fostamatinib increased the rate and extent of absorption of ethinyl estradiol, simvastatin, or rosuvastatin, but had no impact on the pharmacokinetics of warfarin [11] or methotrexate in rheumatoid arthritis patients [12]. Therapeutic effects of fostamatinib in chronic lymphocytic leukemia (CLL) was successfully tested in mice [13] and in clinical trials (phase I and II studies) with proven efficacy and safety in patients with CLL and B cell non-Hodgkin lymphoma (NHL) [2].

Administration of fostamatinib during pregnancy caused urogenital and cardiovascular abnormalities through kidney and ureteral agenesis and a specific major vessel abnormality of the retroesophageal right subclavian artery [14]. Tamatinib displays minimal functional immunotoxicity [15]. In experimental nephrotoxic nephritis in rats, tamatinib reduced glomerular macrophage infiltration, tissue injury, and proteinuria [16]. It also reduced renal monocyte chemoattractant protein glomerulonephritis in a time-dependent manner and improved renal function [16].

Fostamatinib hydrolyzes rapidly to tamatinib by intestinal alkaline phosphatases. It is highly bioavailable with dose-dependent systemic exposure [17]. A study carried out both in vitro and in humans showed that fostamatinib was rapidly transformed to tamatinib by human intestinal microsomes [18,19]. After oral administration of fostamatinib, tamatinib was the major metabolite in plasma while fostamatinib plasma levels were very low [20]. An in-vitro study confirmed that tamatinib is metabolized by CYP3A4 via O-demethylation. Rifampicin (a CYP3A4 inducer) decreased tamatinib blood levels [11]. Concurrent administration of ketoconazole and verapamil with fostamatinib increases tamatinib levels, attributable inhibition of CYP3A4. Fostamatinib lowered concentrations of pioglitazone and its metabolite hydroxy pioglitazone, increased pioglitazone AUC, and decreased production of the hydroxy metabolite. These observations demonstrated that tamatinib is a CYP2C8 inducer [11]. Dose escalation of fostamatinib showed a linear response with rapid onset. In vitro, tamatinib downregulated macrophages stimulated with aggregated IgG and MCP-1 produced from mesangial cells [16].

Tamatinib induces progressive improvement in histopathology, clinical scores, and joint radiography in collagen-induced arthritis in rodents [21]. Tamatinib is used in therapy for glomerulonephritis through targeting of Syk [16] and has shown efficacy and safety in patients with NHL [22]. Tamatinib, as a Syk inhibitor, acts with TGF-β to decrease cell viability. These actions result in a reduction in progression through the cell cycle and increased cell apoptosis [23]. Tamatinib infusion increases blood pressure in anesthetized rats. It inhibits vascular endothelial growth factors dose-dependently [24]. It also increases femoral arterial conductance [5].

Tamatinib, like most TYK inhibitor drugs, has a narrow therapeutic margin. Therefore, individualization of the dose is necessary for safe and efficacious use. Consequently, a sensitive, precise, and accurate method is needed for dose adjustment. There is a lack of fully validated methods for tamatinib determination in plasma. The purpose of this work was to develop an accurate, rapid, and eco-friendly method for detection of tamatinib in rat plasma using UPLC–MS/MS and to apply this method in a pharmacokinetic study.

## 2. Results and Discussion

### 2.1. Optimization of Chromatographic Condition

**Figure 1:**
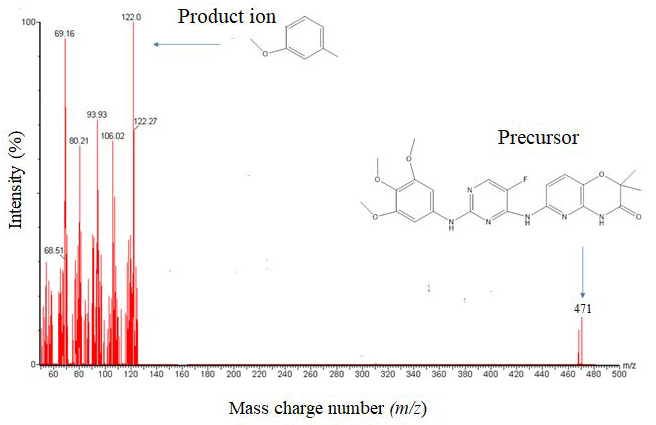
Typical Representation of Precursor-to-Product Ion Spectra of Tamatinib using ESI Positive Ionization Mode.

In chromatographic analysis, the efficiency of analytical methods to separate the analyte and IS depends on its sensitivity, which is represented by LLOD or LLOQ. Both mass spectrometry and chromatographic conditions were optimized. Tamatinib and IS (500 ng/mL) were injected into the mass spectrometer with a flow rate of 5 µL/min. Higher sensitivity for both analyte and IS was obtained using the positive ionization mode. The most abundant fragment ions were found at *m*/*z* 122.0 and 84.0 for the analyte and IS, respectively, and MRM fragmentation of [M+H]^+^ 471.1 > 122.0 and 441.1 > 84.0 were applied for quantitation of the analyte and IS, respectively (Figure 1).

To achieve the best separation, HILIC, Acquity BEH C_18_ and Acquity CSH C_18_ columns were tested. The best column for sensitivity and resolution was Acquity^TM^ (Milford, MA 01757, USA) UPLC CSH C18 (2.1 mm × 100 mm, 1.7 µm). Acetonitrile and methanol were tested as solvents with various amounts of ammonium acetate or ammonium format. The most efficient mobile phase with the best retention times for the analyte and IS was acetonitrile with 10 mM ammonium acetate (90:10) at an elution rate of 0.25 mL/min.

### 2.2. Sample Preparation

Sample preparation is the most important process in a bioanalytical method because it can remove endogenous and other substances to yield higher sensitivity. Protein precipitation, the simplest method in drug analysis, was assessed. However, the recovery or method efficiency was low. This result may reflect suppression arising from interfering endogenous bio-components among the solvents—ethyl acetate, diethyl ether, MTBE, and dichloromethane—tested for extraction of tamatinib and IS. MTBE showed the highest recovery. Finally, a combination of cleanup methods—namely, protein precipitation before liquid–liquid extraction with MTBE—was used to obtain the highest recovery and sensitivity for the analysis of the analyte and IS.

### 2.3. Method Validation

FDA guidelines for bioanalytical methods were followed to validate the present method. The validation comprises of accuracy, precision, extraction recovery, matrix effects, and stability studies. Three concentration levels of QC samples, across the range of CC, were evaluated (LOQ, MQC, and HQC) along with LLOQ.

#### 2.3.1. Selectivity and Sensitivity

**Figure 2:**
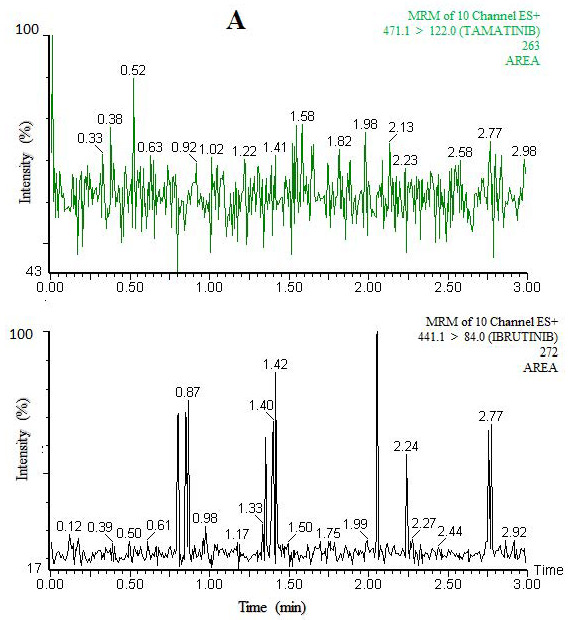

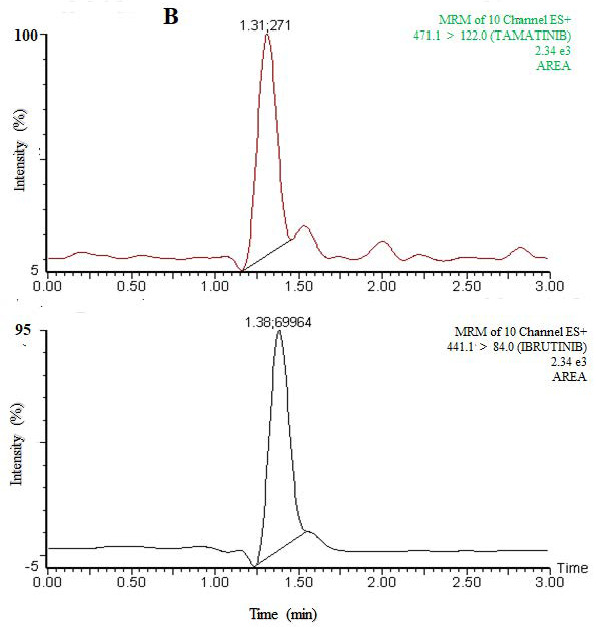
MRM Chromatograms of Tamatinib and Internal Standard in Blank Rat Plasma (**A**), and Plasma Spiked at LLOQ Level (**B**).

No interfering peaks were found at the retention time of tamatinib and IS following analysis of blank plasma extracted using the same extraction and separation method using the optimized UPLC–MS/MS condition. Figure 2 shows a typical chromatogram of the extracted blank rat plasma. Sensitivity, or the relationship between the analyte signal and the concentration, is very important for the quality of the method used for therapeutic drug monitoring and routine analysis, and it is related to the signal-to-noise ratio. To maximize the sensitivity and avoid ionization suppression, mass spectrometry parameters were adjusted to get highest intensity. The values of signal-to-noise ratio were 6.96 and 12.46 for low limit of detection (LLOD) and low limit of quantitation (LLOQ), respectively. The values of these parameters are shown in Table 1.

#### 2.3.2. Linearity

**Figure 3:**
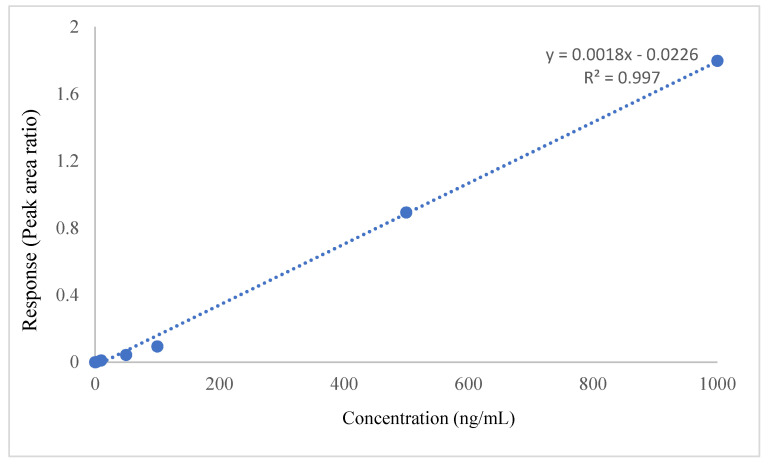
Standard Calibration Curve of Tamatinib.

Linearity was determined by the regression coefficient from a plot of the ratios of tamatinib to IS (analyte/IS) against tamatinib concentration taken from CC. The method’s linearity was within an acceptable range (0.997) (Table 1, Figure 3).

#### 2.3.3. Accuracy and Precision

The LLOQ and three levels of QC were analyzed in five replicates on 3 successive days to determine intra- and inter-day accuracy and precision, respectively. Accuracy is the closeness of a measured value to the actual one or the percentage deviation from nominal concentrations, and precision is expressed as CV%. Accuracy and precision of the method were within acceptable levels. The Guidelines stated accuracy and precision should be ≤±15% and within ±15%, respectively, except for LLOQ, for which they can be ≤±20% and within ±20%, respectively (Table 2).

#### 2.3.4. Recovery and Matrix Effects

The average of recovery and matrix effect were measured by the analysis of QC samples. Mean recovery (method efficiency) was 81.23% among the three levels. Matrix effect was negligible, showing little ion suppression with an average of matrix effect less than 15% (Table 3). The recovery of the other solvents used, such as ethyl acetate, diethyl ether, and dichloromethane, ranged from 55% to 63%.

#### 2.3.5. Stability

Stability of tamatinib was examined under different processing and storage conditions using LQC, MQC, and HQC samples. The results demonstrated that tamatinib was stable in plasma under different conditions, showing percentage of deviation (precision) in all cases of ≤15%. In addition, percentage accuracy of tamatinib was also within the ±15% limit. This indicated that the method was stable during its application and use) Table 4).

### 2.4. Application to a Pha Rmacokinetic Study

**Figure 4:**
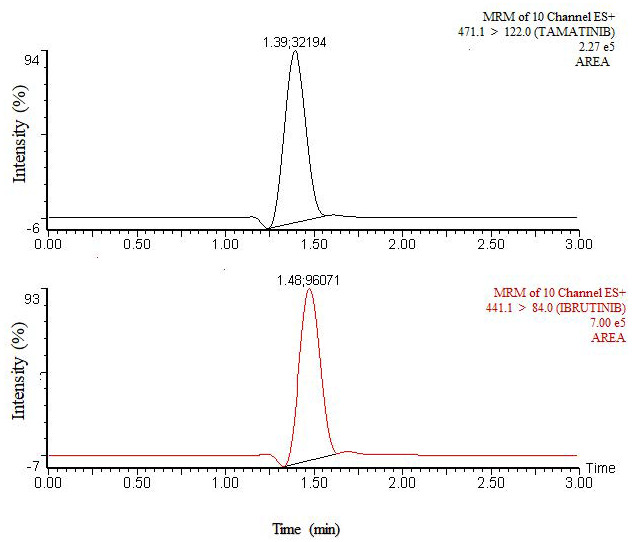
MRM Chromatograms of Tamatinib and Internal Standard in a Real Rat Plasma Sample Obtained at 1.0 h Following Oral Administration of 12.5 mg/kg of Fostamatinib.

**Figure 5:**
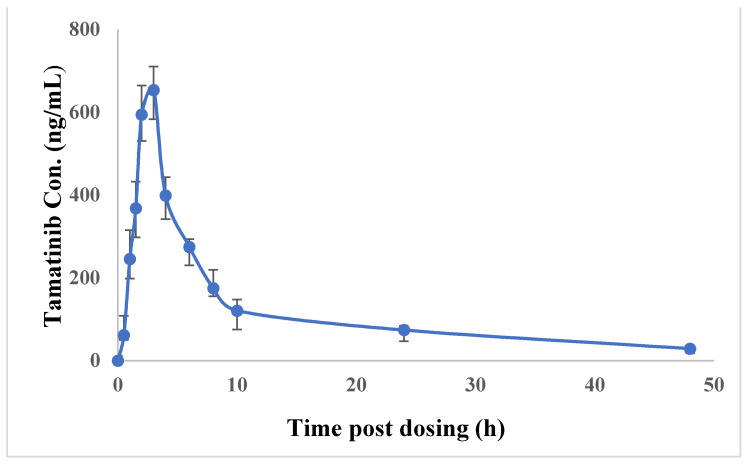
The Time Profile of Tamatinib Concentrations (Mean ± SD) after Oral Administration of 12.5 mg/kg of Fostamatinib.

In pharmacokinetic and clinical studies, fostamatinib was assessed by tamatinib plasma concentrations because the concentration of fostamatinib is low as it is rapidly metabolized to tamatinib [11]. The method was successfully applied to study pharmacokinetics of tamatinib in rats (Figure 4). The average of tamatinib maximum plasma concentration (C_max_) was 653.25 ± 70.4 ng/mL and the corresponding time to reach C_max_ (t_max_) was 3.0 h. The mean area under the time–concentration curve from 0 to 48 h (AUC_0–48h_) was 5644.4 ± 1213.6 ng/mL and AUC_0-inf_ was 6418.5 ± 1495.7 ng/mL. Median residence time (MRT) was 19.77 ± 2.9 h and half-life (t_1/2_) was 18.33 ± 0.9 h. (Table 5, Figure 5). Previously published tamatinib pharmacokinetics studies [7,8,25] used some LC-MS/MS methods. However, these reports did not provide details about their validation methods including drug extraction and separation. Moreover, the sensitivity of our work exceeded the sensitivity in the reported analyses. The current method is characterized by high throughput reflecting short run times with a LLOQ of only 0.1 ng/mL. Moreover, the extraction procedure is simple and quantitative. The method will be advantageous for therapeutic drug monitoring.

### 2.5. Investigation of the Method Greenness

The proposed method fulfills the criteria of the greenness profile according to the emergency planning and community right-to-know act, 2004; Code of Federal Regulations, 2014. None of the used solvents are listed in the PBT list (persistent, bio-, accumulative, and toxic) [26] and are not corrosive, not a hazard. The amount of waste is less than 50 g per sample [27].

**Figure 6:**
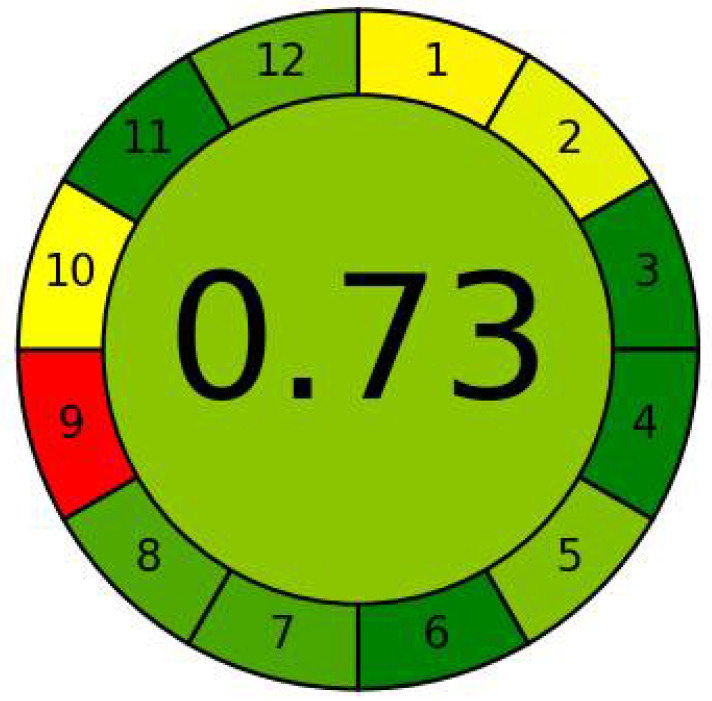
An AGREE Pictogram for Evaluation of the Proposed Method’s Greenness.

The greenness of the analytical method was evaluated using the AGREE green program as well as an analytical eco-scale. The AGREE green program provides information on the whole procedure. The proposed method scored 0.73 point from 1.0, which indicated that the method was good from the ecological point of view (Figure 6). Application of the analytical eco-scale indicated that the proposed method acquired 82 points from 100 (Table 6, Figure S1), and an analytical eco-scale score of more than 50 points represents an good result of green analysis [28].

## 3. Materials and Methods

### 3.1. Chemicals

**Figure 7:**
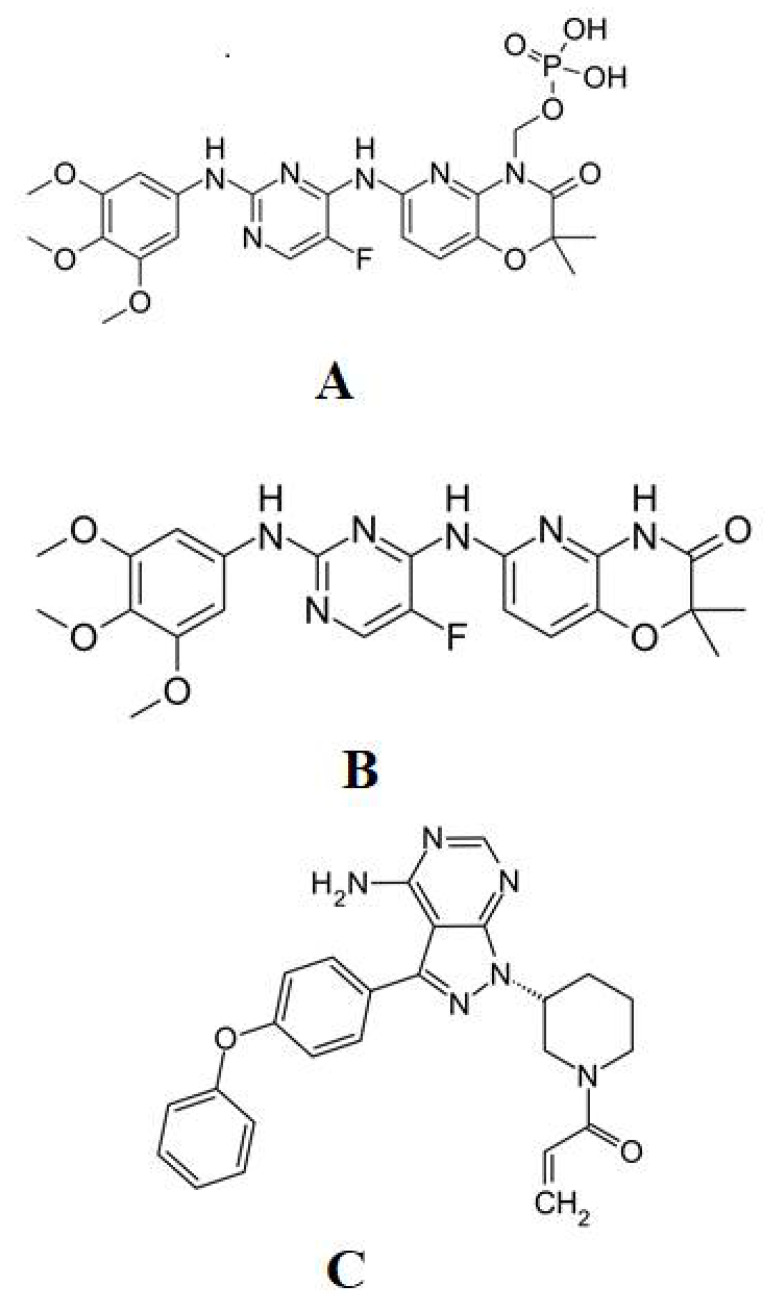
Chemical Structure of Fostamatinib (**A**), Tamatinib (**B**), and Ibrutinib (**C**).

Fostamatinib sodium (Purity, 98%) was purchased from “Toronto Research Chemicals” (North York, ON, Canada), tamatinib (purity; ≥98.0%), ibrutinib (IS, purity ≥97.0%) (Figure 7) was obtained from “Beijing Mesochem Technology Co. Ltd.”. Beijing, China (Figure 7). Methyl tert-butyl ether (MTBE) were obtained from Central Drug Housing Ltd. (New Delhi, India). sulphoxide Acetonitrile and methanol (HPLC grade) were supplied by “BDH Laboratory, Lutterworth, UK”. Dimethyl (DMSO) (Loba Chemie Pvt. Ltd. Mumbai, India), ammonium acetate (Qualikemes Fine Chem. Pvt. Ltd., Vadodara, India) and deionized water from the Milli-Q system Millipore, M (oscheim Cedex, France) were used in the preparation of the buffer of the mobile phase. 

### 3.2. Instrumentation

The analysis was performed on Waters^®^ Acquity H-Class UPLC^®^ tandem triple quadrupole mass spectrometer (TQD) (Waters, Milford, USA). The H-Class UPLC^®^ system contained Acquity sample manager and Acquity quaternary solvent manager. TQD was equipped with electrospray ionization (ESI) probe. Samples were detected and quantified in multiple reaction monitoring (MRM) mode. Chromatographic separation of tamatinib and IS was performed using Acquity TM CSH C18 (2.1 mm × 100 mm, 1.7 µm) column. Mobile phase composition was 10 mM ammonium acetate and acetonitrile (10:90). The mobile phase was adjusted at 0.25 mL/min. The MRM transitions of 471.1 > 122.0 and 441.1 > 84.0 were used for detection tamatinib and IS, respectively. The system was operated with the MassLynx program (Waters corporation, Milford, MA 01757, USA (Milford, MA 01757, USA), and the data acquisition used the TargetLynx^TM^ software. Nitrogen (purity 99.999%) was used as a desolvation gas and argon as a collision gas. MS/MS parameters were optimized for tamatinib and IS by direct infusion of their solutions (500 ng/mL) individually at a flow rate of 5 µL/min. The values of the optimized parameters were illustrated in Table 7.

### 3.3. Preparation of Stock Solutions

Tamatinib was dissolved in DMSO to prepare a stock solution of 4 mg/mL. The solution was diluted 20-fold with methanol to achieve a concentration of 200 µg/mL. An IS working solution of 10 µg/mL was prepared in acetonitrile. The tamatinib working solution was used to prepare calibration curves (CC) and quality control (QC) samples in rat plasma. CC range was 0.1–1000 ng/mL. QC samples were prepared by spiking rat plasma with the tamatinib working solution at low (LQC, 0.3 ng/mL), medium (MQC, 75 ng/mL) and high (HQC, 750 ng/mL) concentrations that covered the entire range of CC.

### 3.4. Sample Preparation

Plasma extraction of tamatinib was carried out using liquid–liquid extraction. In a 2 mL vial, 10 µL of IS (10 µg/mL) was added to 100 µL plasma samples followed by the addition of acetonitrile (50 µL). Samples were vortex-mixed for 30 s, and 1 mL of MTBE was added. All samples were vortex-mixed for 1 min, then centrifuged at 8 °C for 10 min at 4500 g. After centrifugation, the upper layer was transferred to 2 mL tubes and dried using a vacuum concentrator at 40 °C. The residue was reconstituted with 100 µL of the mobile phase and a volume of 5 µL was injected into the UPLC–MS/MS.

### 3.5. Method Validation

The method was validated in compliance with the FDA bioanalytical method validation guidelines [25].

#### 3.5.1. Specificity and Sensitivity

The absence of interference at elution times of the studied drug and IS was evaluated by comparing chromatograms of plasma sample spiked with tamatinib at the lowest concentration (LLOQ) with that of blank plasma obtained from six different rats for any interference at the retention time of tamatinib and IS. According to the signal-to-noise ratio, low limit of detection (LLD) and LLQ can be determined. It should be not less than 5:1 and 10:1 for LLD and LLQ, respectively.

#### 3.5.2. Linearity

Linearity represented by plotting a graph of the relation between tamatinib analyte nominal concentration and analysis response (ratio of the analyte peak area and IS). Linearity was evaluated using CC in rat plasma. CC were prepared with nine different concentrations of tamatinib in the range of 0.1–1000 ng/mL (0.1, 0.5, 2.0, 10.0, 50.0, 100.0, 500.0, 1000.0) and 10 µL of IS was added in the CC samples. Peak area ratios (tamatinib/IS) were plotted against tamatinib concentration. The linearity expressed by correlation coefficient (*r*) was evaluated using weighted (1/*x*^2^) linear regression.

$$r=\frac{n\left(\sum^{\;}xy\right)-(\sum^{\;}x)(\sum^{\;}y)}{\Delta \sqrt{nx^{2}}-\left(\sum^{\;}n\right)n^{2}}$$

#### 3.5.3. Accuracy and Precision

Accuracy and precision of the method was achieved using quality control (QCs) samples. Three QC samples at low, medium, and high concentrations along with LLOQ samples were analyzed. LLOQ should be quantitatively determined with suitable precision and accuracy (±20%) and with a signal more than 5 time the background noise. Six sets of QC samples and LLOQ were analyzed on 3 successive days for evaluation of intra-and inter-day precision and accuracy.

#### 3.5.4. Recovery and Matrix Effect 

The recovery and the matrix effects were determined in rat plasma samples through the anlysis of three QC concentrations (0.3, 75 and 750 ng/mL) level in five replicates. The percentage recovery of analyte was calculated by comparing the percentage of peak area response of plasma samples spiked with analyte before extraction with those fortified after the extraction. The matrix effects were evaluated by post extraction (quantitative) method. The percentage of matrix effects were calculated by comparing the peak area response of plasma samples spiked with analyte after extraction with those of aqueous samples. The same procedure was followed for the evaluation of recovery and matrix effects for IS.

#### 3.5.5. Stability

The stability of tamatinib was studied at different conditions using QC samples at three levels (LQC, MQC, and HQC) in six replicates. Short-term stability was evaluated at 22–25 °C for 6 h, which represented the processing time. Autosampler stability at ambient temperature for 24 h, long-term stability (−80 °C for 6 weeks), and three cycles of thaw and freeze (−80 and 25 °C) were evaluated. The actual concentrations of QCs samples (LQC, MQC, and HQC) under the stability test condition were compared against freshly prepared samples. Moreover, stock solution and working solution stability was evaluated at refrigerator temperature (4 °C) for 2 weeks.

#### 3.5.6. Application of Pharmacokinetic Study

Eight Wistar male rats weighing 210 ± 20 g (aged about 2 months) were provided by National Organization for Drug Control and Research, Cairo, Egypt. Rats were placed in cages in optimum conditions of humidity (40–60%) and temperature (22–26 °C) with regular 12 h day-night cycle. No diet was available for animals for 10 h prior and 1 h post drug administration. Water was available ad libitum. Rats were administered orally (using the gavage technique) 12.5 mg/kg (the middle dose used in the work of Clemens et al. [14]) fostamatinib as a suspension in 1% sodium carboxymethylcellulose [14]. Under light anesthesia, blood samples (0.3 mL) were collected from orbital sinus, in heparinized tube, at different time points; 0, 0.5, 1.0, 2.0, 3.0, 4.0, 6.0, 8.0, 12.0, 24.0, and 48.0 h after drug administration. Plasma samples were obtained by centrifugation at 3000 g for 3 min and kept frozen at −80 °C until analysis. Non-compartmental analysis with the trapezoidal rule was used for estimation of pharmacokinetic parameters. These parameters were calculated using WinNonlin software (Pharsight Co., Mountain View, CA, USA).

The experimental protocol approval was obtained by the ethics committee at the National Organization for Drug Control (NODCAR/VI/47/19).

## 4. Conclusions

In this work, we established a sensitive, fast, reproducible, and reliable method for determination of tamatinib in plasma samples. Tamatinib was extracted by liquid–liquid procedure using MTBE. The matrix effect was negligible and the recovery for both analyte and IS compound were satisfactory. This method showed good linearity with high selectivity, sensitivity, and precision as per FDA guideline. The method was green and eco-friendly, and it was validated and successfully used in tamatinib pharmacokinetic characterization in rats. Therefore, the assay can be used for TDM and pharmacokinetic studies.

## Figures and Tables

**Table 1:** Average of Plasma Standard Calibration Curve of Tamatinib.

Concentration (ng/mL)		Precision	Accuracy
Theoretical	Measured		
(ng/mL)	(Mean ± SD)	(%)	(%)
0.1	0.09 ± 0.01	12.1	87.3
0.5	0.43 ± 0.02	4.7	86.3
2.0	1.77 ± 0.14	7.9	88.3
10.0	8.55 ± 0.41	4.8	85.5
50.0	42.56 ± 2.35	5.5	85.1
100.0	86.43 ± 4.37	5.1	86.4
500.0	489.25 ± 29.53	6.0	97.8
1000.0	872.41 ± 42.5	4.9	87.2

**Table 2:** Inter- and Intra-day Precision and Accuracy of Measurements of Tamatinib in Rat Plasma.

Conc.	Intra-Day			Inter-Day		
(ng/mL)	Mean ± SD	Accuracy	Precision	Mean ± SD	Accuracy	Precision
		(%)	(%)		(%)	CV (%)
0.1	0.085 ± 0.005	85.0	5.8	0.086 ± 0.001	86.0	12.0
0.3	0.255 ± 0.012	85.0	4.7	0.261 ± 0.034	87.0	13.0
75	64.68 ± 2.490	86.2	3.9	66.5 ± 6.720	88.7	10.1
750	648.5 ± 36.000	86.5	5.5	670.5 ± 67.500	89.4	10.1

**Table 3:** Recovery (%) and Matrix Effects for Tamatinib and Internal Standard.

Compound	Nominal Conc. (ng/mL)	Recovery			Matrix Effects		
		Mean	Mean	RSD	Mean	Mean	RSD
		(ng/mL)	(%)	(%)	(ng/mL)	(%)	(%)
Tamatinib	0.3	0.25	83.3	0.01	0.254	84.7	0.02
	75	62.4	83.2	2.79	64.4	85.9	3.37
	750	578.7	77.2	4.2	641.5	85.5	5.6
IS	100	74.1	74.1	6.4	85.93	85.93	5.75

**Table 4:** Stability of Tamatinib in Rat Plasma under Different Storage and Processing Conditions.

Stability	Nominal Concentration (ng/mL)	Measured Concentration (ng/mL)	Precision (%)	Accuracy (%)
Short-term	0.3	0.26 ± 0.03	11.5	86.7
	75.0	64.65 ± 3.47	5.4	86.2
	750.0	640.10 ± 43.19	6.7	85.3
Long-term	0.3	0.26 ± 0.02	7.7	86.7
	75.0	66.10 ± 7.77	11.8	88.1
	750.0	640.60 ± 73.94	11.5	85.4
Thaw and freeze (three cycles from −80 °C to 25 °C)	0.3	0.27 ± 0.01	3.7	90.0
	75.0	67.40 ± 2.79	4.1	89.9
	750.0	649.20 ± 69.95	10.8	86.6
Auto-sampler (24 h)	0.3	0.26 ± 0.02	7.7	86.7
	75.0	64.00 ± 5.11	8.0	85.3
	750.0	654.34 ± 97.10	14.8	87.2

**Table 5:** Pharmacokinetic of Tamatinib Following Administration of 12.5 mg/kg to Rats.

Parameters	Mean ± SD
C_max_ (ng/mL)	653.25 ± 70.4
t_max_ (h)	3.0
AUC_0–48_ (ng/mL)	5644.4 ± 1213.6
AUC_0-inf_ (ng/mL)	6418.5 ± 1495.7
K_el_ (h)	0.04 ± 0.001
t_1/2_ (h)	18.33 ± 0.9
MRT (h)	19.77 ± 2.9
Clearance (L/h)	0.030 ± 0.001
Volume of Distribution (L)	0.019 ± 0.001

**Table 6:** The Penalty Points of the Method for the Determination and Quantitation of the Tamatinib in Plasma.

Parameter	Value	Penalty Points
Dimethyl Sulfoxide	<10 mL (g)	1
Aceonitrile	<10 mL (g)	4
Methanol	<10 mL (g)	6
Formic acid	<10 mL (g)	1
Ammonium acetate	<10 mL (g)	1
Waste	1.8 mL/run (g)	3
Instrument energy	>1.5 kWh	2
Total penalty points		18
Eco-scale score		82

**Table 7:** Mass Optimization Parameters for Tamatinib and Ibrutinib (IS).

Parameters	Tamatinib	Ibrutinib
I. Compound Parameters		
Precursor ion	471.1	441.1
Product ion	122.0	84.0
Dwell time (s)	0.25	0.25
Cone voltage (V)	68	52
Collision energy (eV)	50	46
II. Instrument Parameters		
Collision gas flow rate (L/h)	0.1	0.1
Nitrogen flow rate (L/h)	600	600
Source Temperature (°C)	150	150
Desolvation temperature (°C)	350	350

## Supplementary Materials

The following are available online, Figure S1. Analytical Greenness report sheet and Criteria.

## Sample Availability

Not available.
