# Supplementary material for: Eco-Friendly UPLC–MS/MS Method for Determination of a Fostamatinib Metabolite, Tamatinib, in Plasma: Pharmacokinetic Application in Rats
Source: Molecules. 2021 Jul 31;26(15):4663. doi: 10.3390/molecules26154663 (PMC8348403; doi:10.3390/molecules26154663)
Supplement: Supplementary file 1 [file molecules-26-04663-s001.zip › molecules-1204430-supplementary.pdf]

Analytical Greenness report sheet

29/05/2021 00:24:52

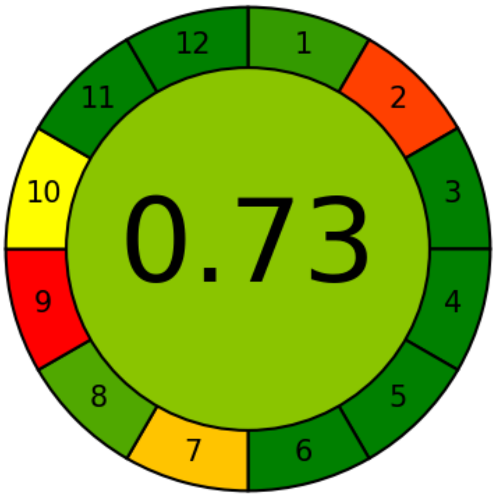

Criteria	Score	Weight
1. Direct analytical techniques should be applied to avoid sample treatment.	0.9	2
2. Minimal sample size and minimal number of samples are goals.	0.13	2
3. If possible, measurements should be performed in situ.	1.0	2
4. Integration of analytical processes and operations saves energy and reduces the use of reagents.	1.0	2
5. Automated and miniaturized methods should be selected.	1.0	2
6. Derivatization should be avoided.	1.0	2
7. Generation of a large volume of analytical waste should be avoided, and proper management of analytical waste should be provided.	0.39	2
8. Multi-analyte or multi-parameter methods are preferred versus methods using one analyte at a time.	0.84	2
9. The use of energy should be minimized.	0.0	2
10. Reagents obtained from renewable sources should be preferred.	0.5	2
11. Toxic reagents should be eliminated or replaced.	1.0	2
12. Operator's safety should be increased.	1.0	2
